# Non-Coding RNAs and Adipogenesis

**DOI:** 10.3390/ijms24129978

**Published:** 2023-06-10

**Authors:** Wenxiu Ru, Sihuan Zhang, Jianyong Liu, Wujun Liu, Bizhi Huang, Hong Chen

**Affiliations:** 1 College of Animal Science, Xinjiang Agricultural University, Urumqi 830052, China; ruwenxiu@nwafu.edu.cn (W.R.); wujunliu1026@xjau.edu.cn (W.L.); 2 Key Laboratory of Animal Genetics, Breeding and Reproduction of Shaanxi Province, College of Animal Science and Technology, Northwest A&F University, Xianyang 712100, China; sihuanzhang1990@ahau.edu.cn; 3 Yunnan Academy of Grassland and Animal Science, Kunming 650212, China; wyf1019@nwafu.edu.cn

**Keywords:** adipogenesis, lncRNA, miRNA, circRNA

## Abstract

Adipogenesis is regarded as an intricate network in which multiple transcription factors and signal pathways are involved. Recently, big efforts have focused on understanding the epigenetic mechanisms and their involvement in the regulation of adipocyte development. Multiple studies investigating the regulatory role of non-coding RNAs (ncRNAs) in adipogenesis have been reported so far, especially lncRNA, miRNA, and circRNA. They regulate gene expression at multiple levels through interactions with proteins, DNA, and RNA. Exploring the mechanism of adipogenesis and developments in the field of non-coding RNA may provide a new insight to identify therapeutic targets for obesity and related diseases. Therefore, this article outlines the process of adipogenesis, and discusses updated roles and mechanisms of ncRNAs in the development of adipocytes.

## 1. Introduction

Adipose tissue is the material basis of living systems, serves as an energy reservoir to regulate caloric balance, and is mainly found beneath the skin (subcutaneous fat), abdominal omentum (omental fat) and surrounding the viscera (visceral fat) [1]. Adipose tissue contains a great amount of mature adipocytes, as well as preadipocytes, mesenchymal cells, and cells within the SVF (stromal vascular fraction) [2]. Generally, adipose tissue can be categorized into white adipose tissue (WAT), brown adipose tissue (BAT) and beige adipose tissue. Contrary to white adipocytes with storing energy, brown and beige fat cells are capable of thermogenesis, and thus to expend energy [3]. These adipocytes are characterized by an increase in mitochondria and possess numerous small lipid droplets. Meanwhile, uncoupling protein 1 (UCP1) has been regarded as the key factor that drives thermogenesis [4]. For the purpose of this review, we mainly focus on white adipose. Besides acting as a vital energy reservoir, adipose tissue is considered an endocrine organ. Signaling proteins secreted by white adipose tissue are known as adipokines, including adiponectin, leptin, tumor necrosis factor (TNF), interleukin 6 (IL-6), adipocyte fatty acid-binding protein (AP2) and so on, which are involved in coordinating the body’s metabolism [5].

It is important to control the enlargement of existing adipocytes (hypertrophy) and the formation of new adipocytes (hyperplasia) for the metabolic health of the body [4]. For example, obesity, as a chronic disease, results from excessive fat deposition and abnormally secreted adipokines. However, for livestock, ectopic fat deposition such as intramuscular fat is the key to improving the meat quality. The past few years have expanded our understanding of fat formation and what regulating factors are involved. Recently, many research studies have shifted their attention to the epigenetic mechanisms in regulating adipogenesis, especially noncoding RNAs (ncRNAs). ncRNAs mainly include long noncoding RNAs (lncRNAs), microRNA (miRNA), circular RNA (circRNA), small nuclear RNA (snRNA), and others [6]. Most of the past research has uncovered the roles of lncRNA, miRNA, and circRNA in the regulation of adipogenesis. Therefore, this article outlines the process of adipogenesis, and discusses how ncRNAs influence the development of adipocytes.

## 2. Process of Adipogenesis

**Figure 1:**
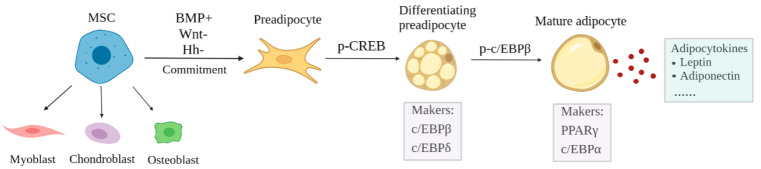
The process of adipogenesis.

At present, there is a relatively clear description of adipogenesis. Adipocytes arise from multipotent mesenchymal stromal cells (MSCs), which have the ability to develop into multiple cell types including adipocytes, chondrocytes, myocytes, and osteocytes [7]. For adipogenesis, MSCs acquire adipogenic fates from specific signaling molecules to develop into preadipocytes, and then experience mitotic clonal expansion (MCE) and terminal differentiation to form mature adipocytes under the regulation of key transcription factors [8] (Figure 1). A series of extracellular signaling pathways are essential for adipocyte commitment of MSCs, mainly including hedgehogs (Hh), wingless-type MMTV integration site (Wnt) signaling, and bone morphogenic protein (BMP) signaling [9]. Here, BMP2, BMP4, and BMP7 could commit MSCs to the adipogenic lineage through activating downstream signaling Smad and p38/MAPK [10,11]. On the contrary, Wnt and Hh signaling has been extensively confirmed as a negative regulator of adipogenesis [3]. For determination of mesenchymal lineage, Wnt signaling promoted chondrocyte, myocyte, and osteocyte lineage commitment, while adipogenesis is the only process that is inhibited by Wnt [12]. Wnt signaling has been shown to inhibit the terminal differentiation of preadipocytes into mature adipocytes [13]. For example, WNT10B can inhibit the adipogenic transcription factors peroxisome proliferator-activated receptor γ (*PPARγ*) and CCAAT/enhancer binding protein α (*C/EBPα*) to maintain preadipocytes in an undifferentiated state [14]. Hh signaling can activate a series of intracellular signals leading to regulated expression of target genes through the transcription factor Gli family, including Gli1, Gli2, and Gli3 [3]. During the adipogenic differentiation of MSCs, Hh signaling is weakened due to decreases in Gli expression [9]. The activation of Hh signaling could impair adipogenesis and block the lipid accumulation through interfering the expression of *C/EBPα* and *PPARγ* [15]. It is worth noting that these signaling molecules are synergistic to determine the lineage commitment of MSCs.

Following commitment, several signaling molecules have a crucial effect on adipogenic differentiation. Insulin-like growth factor 1 signaling (IGF-1), glucocorticoid signaling (GC), and cyclic AMP signaling (c-AMP) have a positive role in adipogenesis [16,17,18], while the Notch signaling pathway shows an inhibitory role [19]. Therefore, IGF1, glucocorticoid, and cAMP as differentiation inducers could trigger preadipocytes entering into a series of differentiation programs, including mitotic clonal expansion (MCE) and differentiation cascade [7]. Numerous adipocyte-specific transcription factors were involved in transcriptional differentiation cascade, including cAMP response element-binding protein (CREB), CCAAT enhancer-binding protein α, β and δ (C/EBPα, C/EBPβ, and C/EBPδ), and peroxisome proliferator-activated receptor gamma (PPARγ) [20]. The phosphorylation of *CREB* is thought to be the initiation to these events, and active *CREB* can promote the expression of endogenous *C/EBPβ* and *C/EBPδ* [21,22]. Inactive *C/EBPβ* is phosphorylated by MAP kinase and GSK3β to possess DNA binding activity, and then the active *C/EBPβ* induces the transcription of *PPARγ* and *C/EBPα* [23]. Once expressed, *PPARγ* and *C/EBPα* coordinately trigger the transcription of adipocyte genes to produce the adipocyte phenotype [9,24]. Mature adipocytes are blessed with the appearance of adipocyte and metabolic characteristics, such as the accumulation of triglycerides in the cytoplasm [7]. Furthermore, a large number of regulatory proteins, including cytokines, enzymes, and peptide hormones, can be excreted by adipocytes [25]. The majority of adipocytokines act locally through paracrine, while others, just like leptin and adiponectin, have long-range impacts [26,27].

## 3. LncRNAs and Adipogenesis

### 3.1. LncRNA-Mediated Adipogenesis

LncRNAs are defined as a category RNA transcript greater than 200 nucleotides, and were initially regarded as non-coding genes [28]. However, recent studies have indicated that there are lncRNAs with the capacity to encode small polypeptides in some cases [29,30]. Likewise, lncRNAs are also characterized with 5′ cap and 3′ polyadenylation, and can produce different transcripts through variable shearing [31]. Unlike mRNA, lncRNAs possess poor conservatism among species and have obvious tissue or cell specificity [32]. LncRNAs are located in the cytoplasm and nucleus, but are more abundant in the nucleus [33]. Importantly, lncRNAs play vital roles in various biological processes through affecting chromosome modification, transcriptional activation, miRNA binding, mRNA translation, and protein stability [34].

Recently, numerous lncRNAs related to adipose development have been identified using high-throughput sequencing technology, and the majority are characterized by adipose-tissue-specific expression. Sun and his colleagues found that 175 lncRNAs are specifically regulated by key transcription factors including *PPARγ* and *CEBPα* during adipogenesis. Importantly, deletion of identified functional lncRNAs resulted in a reduction in enrichment for adipose-associated genes, and decreased fat accumulation [35]. Moreover, more adipogenesis- and development-related lncRNAs were screened in humans, mice, bovines, pigs, and so on [36,37,38,39]. SRA (steroid receptor RNA activator) was firstly identified as a lncRNA which had a regulatory role in adipogenesis. Studies have shown that SRA promotes 3T3-L1 adipocyte differentiation through binding *PPARγ* and increasing the expression of *PPARγ* [40]. Other lncRNAs have also been found to the modulate transcriptional activity of *PPARγ*, such as IMFNCR, ADNCR, and Plnc2 [41,42,43]. Similarly, there are some lncRNAs specifically regulating *C/EBPα* [44]. For example, *C/EBPα* activated the expression of lncRNA TINCR through binding its promoter, and TINCR adsorbed miR-31 to promote the transcriptional activity of *C/EBPα*, which formed a TINCR-miR-31-*C/EBPα* feedback loop to enhance adipogenic differentiation in adipose-tissue-derived mesenchymal stem cells (ADSCs) [45]. In addition to being involved in adipocyte differentiation, several lncRNAs have also been implicated in preadipocyte proliferation. For instance, lncAcart promotes 3T3-L1 cell proliferation [46]; lncPRDM16 inhibits preadipocyte proliferation in chickens [47]; lncFAM200B suppresses preadipocyte proliferation in cattle [48]. Antisense lncRNAs (AS lncRNAs), a specific subclass of lncRNAs, are transcribed from DNA on the opposite strand of mRNA. Studies have shown that PU.1 AS lncRNA prevents *PU.1* mRNA translation through binding *PU.1* mRNA to form an mRNA/AS lncRNA compound, which can promote adipogenesis [49,50]. Furthermore, some lncRNAs have negative regulatory effects on adipose development. During the commitment of BMSCs (bone marrow mesenchymal stem cells) into adipocytes, lncRNA H19 inhibited adipogenic differentiation [51]. Likewise, adipoQ (adiponectin) AS lncRNA was found to inhibit adipogenesis [52]. Recently, an increasing number of studies have revealed adipogenesis- and development-related lncRNAs (Supplementary Table S1). It follows that lncRNAs can function as effective regulatory factors to determine adipogenesis and development.

### 3.2. The Regulatory Modes of LncRNAs in Adipogenesis

**Figure 2:**
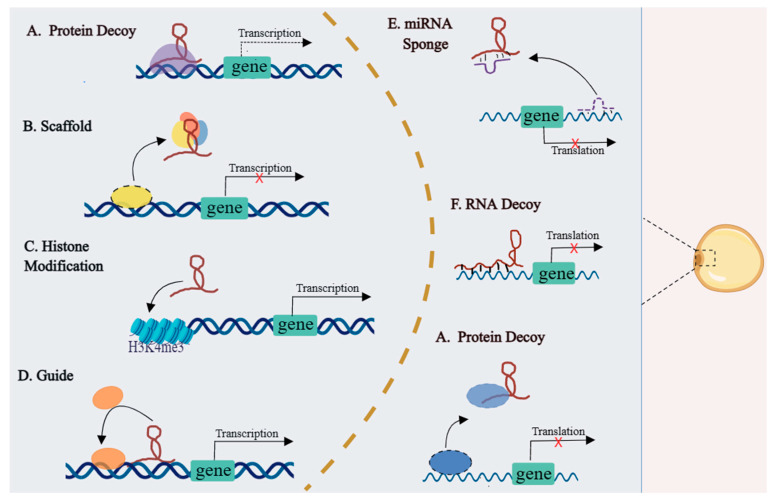
Schematic diagram of the regulatory mechanisms of lncRNA in adipogenesis. The nuclear lncRNAs are involved in modulating gene transcription through acting as protein decoys, scaffolds, guides, and regulation of histone modification. The cytoplasmic lncRNAs mainly affect mRNA stability and translation through functioning as miRNA sponges, RNA, and protein decoys.

To make better sense of how lncRNA participates in adipogenesis and development, it is necessary to discuss the regulatory pathways of identified lncRNAs. The functional mechanisms of lncRNAs are mostly dependent on cellular localization. Nuclear lncRNAs are mainly involved in modulating gene expressions [53,54], while cytoplasmic lncRNAs mainly regulate mRNA stability, translation, and protein phosphorylation [55]. We next turn to summarize identified regulatory pathways of lncRNAs in adipogenesis and development (Figure 2). A major mode of lncRNAs regulating transcription is acting as molecular decoys, including binding RNA-binding proteins (RBPs), RNA molecules, or miRNAs [56]. LncRNA CAAlnc1 bound with Hu antigen R (HuR) to inhibit adipogenesis in C3H10 cells, which was due to the combination of HuR with CAAlnc1 to block the transcription of *C/EBPα* and *PPARγ* [57]. Recently, research found that lncRNA XIST promoted brown preadipocyte differentiation and prevented high-fat-diet-induced obesity through binding with *C/EBPα* [58]. Likewise, lncRNAs bind with RNA molecules to modulate adipogenesis, such as AS lncRNA PU.1 promotes adipogenesis through binding *PU.1* mRNA and *adipoQ* and inhibits adipogenesis through preventing adiponectin mRNA translation [49,52]. The widely studied and best understood regulatory mechanism of lncRNAs is acting as miRNA decoys. LncRNA-Adi promote adipogenesis in adipose-tissue-derived stromal cells (ADSCs) through blocking the interaction with miR-449a and *CDK6* to enhance *CDK6* translation [59]. Moreover, there are more lncRNAs as miRNA decoys involved in regulating adipogenesis, such as when lncRNA Gm15290 promotes fat deposition by sponging miR-27b in mice [60]; lncADNCR inhibits adipogenic differentiation by sponging miR-204 in bovines [42]; and lncIMF2 accelerates adipogenesis by sponging miR-217 in pigs [61]. Generally, proteins are considered the main elements in a variety of scaffolding complexes. However, recent studies indicated that lncRNAs may also serve as players in the scaffold [56]. In BMSCs, Bmncr accelerated assembly of the RUNX2/PPARG and TAZ transcriptional complex by acting as a scaffold for TAZ and ABL, thereby inhibiting adipogenesis and promoting osteogenesis [62]. LncRNAs can also regulate adipogenesis via histone modifications. The missing MIR31HG inhibits adipocyte differentiation through reducing the enrichment of histone H3 lysine 4 trimethylation (H3K4me3) and acetylation (AcH3) in the promoter of *FABP4* [63]. In addition, lncRNAs can regulate gene expression in cis (on neighboring genes) or trans (distantly located genes) ways through guiding the ribonucleoprotein complex to specific targets [56]. LncADINR, lying in ∼450 bp upstream of the *C/EBPα* gene, specifically binds with PA1 and recruits the MLL3/4 histone methyltransferase complex to activate the transcription of *C/EBPα* in *cis*, which promotes adipogenesis [64]. For trans mechanisms, the interaction of slincRAD and DNMT1 can guide epigenetic factors to regulate promoter methylation of cyclin-dependent kinase inhibitor *p21*, which mediates early adipogenesis [65]. As regards lncRNA regulation of splicing, a study found that Ctcflos mediated alternative splicing of *Prdm16* (a key browning factor) during thermogenic adipogenesis [66]. In addition, a few studies have shown that lncRNAs have the capacity to produce functional small peptides, such as LINC00961-encoded SPAR polypeptide, which was identified to regulate muscle regeneration [29]. However, the study of lncRNA-encoding functional peptides during adipogenesis has not been reported, and further research needs to be explored.

## 4. MiRNAs and Adipogenesis

### 4.1. MiRNA-Mediated Adipogenesis

MiRNAs are defined as a class of short ncRNAs with ~23 nucleotides, which function in the post-transcriptional regulation of genes [67]. MiRNAs are evolutionarily conserved and have tissue specificity [68]. The transcription of miRNA was initially mediated by RNA polymerase II (Pol II) to produce primary miRNAs (pri-miRNAs). Subsequently, pri-miRNAs were cleaved to precursor miRNAs (pre-miRNAs) with a microprocessor including RNase III enzyme Drosha, DiGeorge critical region 8 (DGCR8), and other auxiliary factors. Pre-miRNAs were transported into the cytoplasm and processed by the endoribonucleases Dicer to miRNA duplex intermediate. Finally, one miRNA strand was selected by the Argonaute (AGO) protein as mature miRNA, and then assembled RNA-induced silencing complex (RISC) to mediate gene silencing [69]. In general terms, miRNA binds directly to the 3′ untranslated region (3′ UTR) of mRNAs involved in mRNA decay and translation repression. The positions 2~8 of the miRNA, called the seed region, possess strong complementarity with the 3′ UTR of mRNAs [68]. Notably, almost 60% of mammalian transcripts can be targeted by miRNA, and individual miRNA can target multiple mRNAs [67]. 

MiRNAs have become extensive regulators in various biological processes and are involved in almost every cellular process [67]. Likewise, miRNAs also play vital roles in adipocyte development and function. One study has showed that disruption of miRNA processing by fat-specific knockout Dicer in mice can lead to a decrease in subcutaneous and intra-abdominal white fat and impaired metabolic function, which suggested the global importance of miRNAs in adipogenesis [70]. In addition, most studies identified differentially expressed miRNAs at various stages of adipose development through miRNA sequencing. Using microarray analysis, 386 differentially expressed miRNAs were examined during 3T3-L1 differentiation (0, 1, 4, and 7 d after adipogenic induction) and several of these, such as let-7, miR-143, miR-193, miR-103, and miR-210, were increased after 2 d differentiation and maintained high expression levels in mature adipocytes [71]. Another study examined differentially expressed miRNAs during the three different stages of adipogenesis, including human mesenchymal stem cells (hMSCs), human visceral preadipocytes (vHPA), and mature adipocytes, and the results showed that miR-146b exhibited a higher level in mature adipocytes, whereas it exhibited lower expression in vHPA and hMSCs [72]. MiRNAs have also been identified to be involved in the regulation of the process of adipogenesis, including adipocyte commitment of MSCs, MCE, and terminal differentiation [73]. For example, miR-204/211 promoted adipogenesis of mesenchymal progenitor cells and BMSCs through inhibiting the expression of *Runx2*, a key transcription factor for osteogenesis [74]. Using the high throughput microarray, 47 miRNAs were identified to regulate adipogenesis by influencing WNT signaling, of which miR-210 can inhibit WNT signaling by targeting *Tcf7l2*, and thus promote adipogenesis [75]. On the other hand, let-7 had a significant effect on the transition from clonal expansion to terminal differentiation, in which let-7 inhibited clonal expansion through targeting *HMGA2* [71]. Several miRNAs have also been shown to regulate terminal differentiation of adipocytes. For example, miR-143, miR-378, and miR-199 promoted adipocyte differentiation [76,77,78]; in contrast, miR-27a/b, miR-448, and miR-138 inhibited adipocyte differentiation [79,80,81,82]. Furthermore, miRNA mediated adipogenesis by regulating preadipocyte proliferation. MiR-152 has been proven to inhibit preadipocyte proliferation and promote lipid accumulation in 3T3-L1 [83]. There are several miRNAs that have been shown to be involved in the regulation of mature adipocyte metabolic functions [73]. For example, overexpression of miR-519d disrupts fatty acid metabolism and promotes adipocyte hypertrophy, and the overexpression of miR-124a leads to a decrease in lipid metabolism and the accumulation of cellular triacylglycerol (TG) [84,85]. Recently, an increasing number of studies have revealed adipogenesis- and development-related miRNAs (Supplementary Table S2). These findings have elucidated the crucial roles of miRNAs in adipogenesis and metabolic function.

### 4.2. The Regulatory Modes of MiRNAs in Adipogenesis

**Figure 3:**
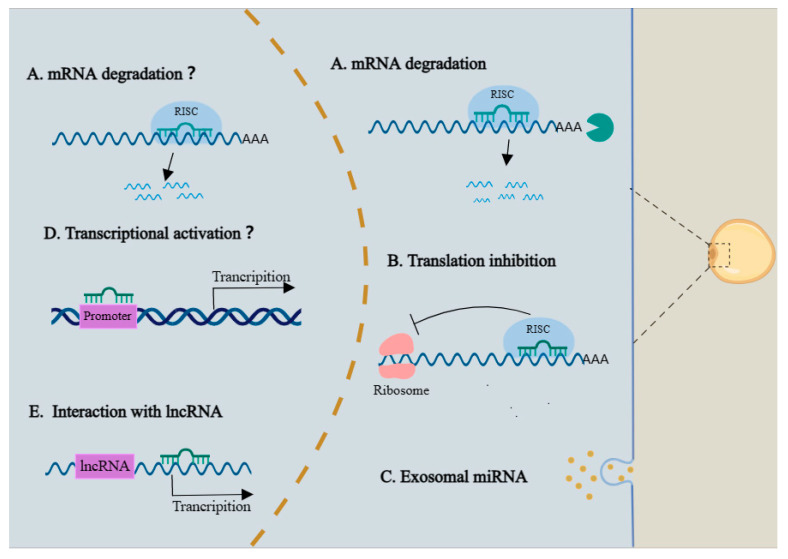
Schematic diagram of the regulatory mechanisms of miRNA in adipogenesis. The cytoplasmic miRNAs mainly mediate mRNA decay and translation repression through binding with target genes. MiRNAs can also be excreted from adipocytes as exosomes. The nuclear miRNAs can mediate gene silencing via RISC, as well as activate gene transcription through binding with promoter. However, these nuclear functions of miRNAs have not been reported in adipogenesis. In addition, the nuclear miRNAs can regulate lncRNA expression.

It is well-known that the regulatory mechanisms of miRNAs on gene expression are principally in combination with the target mRNAs, and thus to mediate mRNA decay and translation repression. We next turn to summarizing the identified regulatory pathways of miRNAs in adipogenesis and development (Figure 3). During adipogenesis, miRNAs can regulate adipogenesis by acting on key transcription factors or signaling pathways associated with adipogenesis. PPARγ is a major transcription factor for adipogenesis whose 3′ UTR sequences are highly conserved, so several miRNAs have been identified to regulate the expression of *PPARγ*. For example, miR-27a, miR-27b, and miR-130 negatively regulated adipocyte differentiation through binding *PPARγ* 3′ UTR [81,82,86]. In addition, miR-31 were found to bind *C/EBPα*, and thus to inhibit adipocyte differentiation [87]. Some miRNAs positively regulated adipogenesis by targeting anti-adipogenic factors, such as miR-146b targets *KLF7* and miR-103 targets *MEFD2* [72,88]. MiR-143 can modulate MAP2K5-ERK5 signaling through targeting the 3′ UTR of *MAP2K5* to involve in adipogenesis [89]. Although most miRNAs binding sites are thought to be located in the 3′ UTR of mRNAs, some studies have shown that miRNAs can also bind to the 5′ UTR or even coding regions of mRNAs, thereby regulating gene expression [90,91]. For instance, miR-130 inhibited adipogenesis by targeting both the coding regions and the 3′ UTR of *PPARγ*, and miR-23a/b-3p accelerated hepatic lipid accumulation by binding the 5′ UTR of *Srebp-1c* and *Fas* [86,91]. It should be noted that the combination between miR-23a/b-3p and the 5′ UTR of targeted genes increased mRNA stability [91]. Besides binding with mRNA, miRNA can interact with lncRNA. One example of this is that miR-140, localized in the nucleus, bound with NEAT1 to enhance its expression, which resulted in increased adipogenesis [92]. Additionally, miRNA-378/378* was found to increase C/EBPα and C/EBPβ activity on the *GLUT4* promoter, thus stimulating lipogenesis [77]. MiRNAs can also be secreted in the way of exosomes to establish a communication network between cells, which can regulate the function of cells and tissues [93]. In this context, adipose tissue has been identified as a major source of exosomal miRNAs, and adipose-derived exosomal miRNAs can produce far-reaching systemic effects through regulating gene expression in other tissues [94]. Likewise, other tissue-derived exosome miRNAs are blessed with an ability to influence adipocyte function. Recent studies, for example, showed that hepar-derived exosomal miR-130a-3p increased the glucose uptake in adipocytes, and ischemic heart-derived exosomal miR-23-27-24 cluster impaired endocrine function in adipocytes [95,96]. Several nuclear functions of miRNAs have been also described, including regulation of the stability of nuclear transcripts, mediating alternative splicing events, interaction with gene promoter regions to modulate gene transcription, binding with gene enhancer sequences to activate transcription, and so on [97,98]. However, nuclear functions of miRNAs have not been fully revealed in adipocytes, and more studies are needed to fully explore the new regulatory modes of miRNAs in adipogenesis.

## 5. CircRNAs and Adipogenesis

### 5.1. CircRNA-Mediated Adipogenesis

CircRNA, as an emerging type of ncRNA, is the covalently closed transcript formed by the back-splicing of mRNA. According to circularization mechanisms, circRNAs are normally classified into ciRNA composed of introns, EcircRNA composed of exons, and EIciRNA composed of introns and exons [99]. At present, three popular models were proposed to account circRNA biogenesis, including the lariat-driven model, the RBP-driven model, and the intron-pairing driven model [100,101]. Intron-pairing-driven cyclization is the most common, in which flanking introns containing reversely complementary sequences, such as the Alu sequence, can promote circRNA formation [101]. Recent studies have revealed circRNA characterization. CircRNAs are evolutionarily conserved, and their expressions are tissue- or developmental-stage-specific [102]. CircRNAs are ubiquitous in various eukaryotes, and circRNAs are more abundant than corresponding linear mRNAs in situations [101]. Additionally, the covalently closed-loop structure of circRNA makes it resistant to degradation by RNases, and thus is more stable than linear transcripts [103]. Recently, numerous circRNAs have been identified, and their functional roles in various biological processes have been uncovered.

CircRNAs are also vital regulators in adipocyte development and function. Abundant adipose circRNAs were identified via the deep sequencing of visceral and subcutaneous fat, many of which were dynamically changed during adipogenesis [104]. Importantly, identified circArhgap5-2 exhibited the essential role of promoting the global transcriptional program of adipocyte genes, and this function of circArhgap5-2 is consistent in human adipocytes [104]. Likewise, more circRNAs associated with adipogenesis were identified in livestock, such as bovines, pigs, chickens, and so on, which can affect fat deposition, and thus improve meat quality [105,106,107]. For example, circPPARA identified in pigs can promote differentiation of porcine intramuscular preadipocytes, thereby increasing intramuscular adipogenesis [105]. The circRNA microarrays analysis for adipose tissue from lean and obese individuals revealed that circSAMD4A was particularly upregulated in obese individuals, and it can promote adipogenesis through the miR-138-5p/*EZH2* axis [108]. Recent studies demonstrated that circRNAs are also involved in adipocyte commitment of MSCs [109]. Circular RNA CDR1as can absorb miR-7-5p to enhance *Wnt5b* expression, thereby promoting adipogenic differentiation and inhibiting osteogenesis in BMSCs [110]. Moreover, circRNA can indirectly regulate the expression of key transcription factors associated with adipogenesis. For instance, newly identified bovine circRNF111 enhanced the *PPARγ* transcription by relieving the inhibitory effect of miR-27a-3p on *PPARγ*, resulting in increased adipogenesis [111]. On the other hand, circRNAs have also been implicated in preadipocyte proliferation. Novel circPPARA has been proven to promote differentiation and inhibit the proliferation of porcine intramuscular preadipocytes [105]. Another work identified that circBDP1 accelerated the proliferation and differentiation of bovine preadipocytes [112]. An increasing number of studies have revealed adipogenesis- and development-related circRNAs (Supplementary Table S3). However, studies on circRNAs’ regulation of adipogenesis are still in their infancy; numerous circRNAs involved in adipogenesis remain to be discovered.

### 5.2. The Regulatory Modes of CircRNAs in Adipogenesis

**Figure 4:**
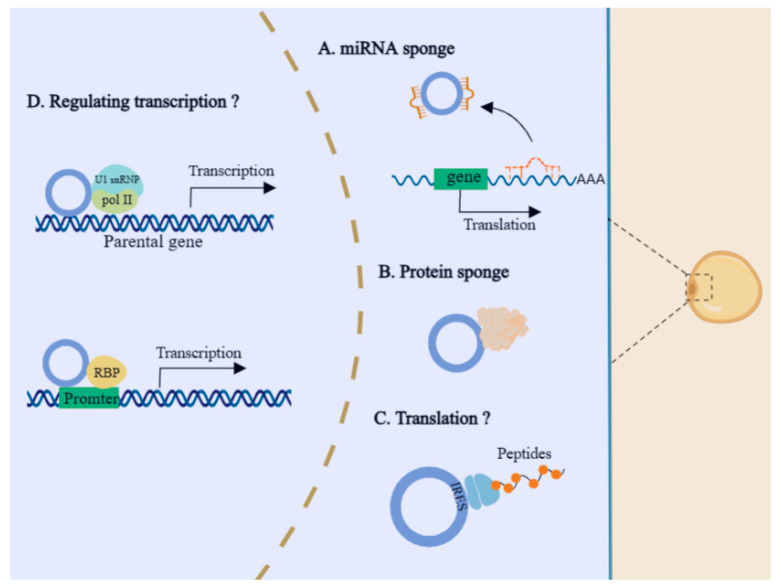
Schematic diagram of the regulatory mechanisms of circRNA in adipogenesis. The cytoplasmic circRNAs exert their functions mainly through acting as miRNA sponges, protein sponges. The nuclear circRNAs regulate gene transcription by interacting with Pol II and U1 snRNP, as well as binding with RBP. In addition, some circRNAs can be translated into peptides. However, regulatory mechanisms in the nucleus and translation function of circRNAs have not been reported in adipogenesis.

Recent studies have proposed the regulatory mechanisms of circRNAs in gene expression, mainly including action as miRNA sponges, regulation of variable splicing, interaction with RBPs, and translation into proteins [113]. We next turn to summarizing the identified regulatory pathways of circRNAs in adipogenesis and development (Figure 4). The functional mechanisms of circRNAs are also mostly dependent on cellular localization. Like lncRNA, cytoplasmic circRNAs are widely regarded as miRNAs decoys. During adipogenesis (preadipocyte, differentiating preadipocyte, and mature adipocyte), 41 differentially expressed circRNAs were identified with RNA-seq, and numerous circRNAs possessed miRNAs binding sites, such as miR-30c, miR17, and miR-130, which suggested that these circRNAs may be involved in the regulation of adipogenesis through potentially sponging miRNAs [114]. Increasing numbers of research studies have elucidated this mechanism. In humans, circSAMD4A promotes preadipocyte differentiation by decoying miR-138-5p to increase *EZH2* expression [108]; in cattle, circFUT10 inhibits adipocyte differentiation by sponging let-7 to increase *PPARGC1B* expression [115]; in pigs, circSETBP1 accelerates adipocyte differentiation by acting as an miR-149-5p sponge [116]. Recent studies have reported that circRNA regulated adipogenesis via an interaction with protein. It was found that deletion of circH19 enhanced adipogenic differentiation in human adipose-derived stem cells (ADSCs). Mechanically, deletion of circH19 inhibited the interaction with PTBP1, and accelerated the effect of PTBP1 on SREBP1 cleavage and translocation, resulting in increased expression of lipogenesis-related genes and accumulated lipids in hADSCs [117]. CircRNAs can also be secreted by adipocytes, and in the way of exosomes to regulate the function of cells and tissues. For example, exosome circ-DB secreted from adipocytes can accelerate tumor growth by sponging miR-34a and activating deubiquitination-related USP7 [118]. Furthermore, nuclear circRNAs are mainly involved in modulating gene transcription and alternative splicing [119]. For example, EIciRNAs can promote the expression of parental genes by interacting with RNA polymerase II, U1 small nuclear RNA, and gene promoters [120]. However, the intracellular mechanisms of circRNAs regulating gene transcription in adipogenesis have not been reported, and more studies are needed to fully explore this.

## 6. Conclusions

Adipogenesis is regarded as an intricate process in which multiple regulatory factors and signal pathways are involved. At present, emerging studies demonstrated that ncRNAs as regulators of gene expression play crucial roles in adipogenesis and development. Therefore, we summarized the previous work to indicate a wide range of roles of ncRNAs, mainly including lncRNA, miRNA, and circRNA in adipogenesis.

With advances in high-throughput sequencing and molecular techniques, an increasing number of adipose ncRNAs are revealed and their functions are actually well established. However, the regulatory mechanisms of ncRNAs modulating adipogenesis in existing reports are still unitary. Previous research mainly focused on the functions of ncRNA as a sponge, especially as an miRNAs decoy. For instance, the mechanism by which nuclear circRNA and miRNA regulates gene transcription in adipogenesis has not been reported. On top of that, further studies are needed to fully reveal the regulatory mechanism of these. In addition, the combined effects of ncRNAs should also be considered. LncRNAs can sponge miRNAs to regulate the expression of target genes. In the same way, circRNA can also directly adsorb miRNAs to relieve the inhibitory effect on genes. The interaction network of lncRNA, miRNA, and circRNA is a potentially crucial regulatory mechanism in cells, and may produce considerable effects on adipogenesis. On the other hand, lncRNA, miRNA, and circRNA can be secreted in the way of exosomes to established a communication network between cells, which can regulate the function of cells and tissues. Currently, exosomal ncRNAs have been described as a potential biomarker of disease [121]. These findings provided a promising strategy for ncRNAs’ action as a diagnostic tool in lipid-related diseases like obesity.

Recently, a popular term of adipose-tissue plasticity has been proposed with the deepening of understanding of adipose tissue, which means that adipose tissue could change its metabolism, structure, and phenotype to meet the needs of the organism in physiologic stimuli [122]. Studies suggests that the limitation in adipose-tissue plasticity leads to the abnormal functions and metabolism of adipose tissue, and drives the progression of lipid-related diseases like obesity. In this context, ncRNAs are emerging as new regulators in adipose-tissue plasticity. At present, the roles of ncRNAs in modulating adipose-tissue plasticity are mainly embodied in phenotype, including regulation of the browning of white fat. For example, lncBATE10 acts as a positive regulator of full brown fat differentiation and white fat browning program [123]; miR-30b/c promoted thermogenesis and the browning process of WAT [124]. Therefore, the potency of ncRNAs as regulators for the abundance and activity of adipose tissue remain, which will yield a promising strategy to combat obesity. However, studies on ncRNA-mediated phenotype plasticity are still in their infancy, and in particular, the research on metabolism plasticity and structure plasticity remain limited. 

Overall, the adipose regulatory network centered on ncRNAs is sketched out in a rough frame. Numerous ncRNAs associated with adipogenesis and their functions and regulatory mechanisms still need to be further specified in more depth.

## Data Availability

Not applicable.

## Supplementary Materials

The following supporting information can be downloaded at: https://www.mdpi.com/article/10.3390/ijms24129978/s1
