# Supplementary material for: Non-Coding RNAs and Adipogenesis
Source: Int J Mol Sci. 2023 Jun 10;24(12):9978. doi: 10.3390/ijms24129978 (PMC10298535; doi:10.3390/ijms24129978)
Supplement: Supplementary file 1 [file ijms-24-09978-s001.zip › ijms-2413305-supplementary.pdf]

Supplement Table S1

LncRNAs involved in adipogenesis and development

LncRNA	Cell	Function	Year
TINCR	ADSCs	Promote adipogenic differentiation	2018
HoxA11-AS1	ADSCs	Promote adipogenic differentiation	2018
Bmncr	BMSC	Inhibit adipogenesis	2018
Plnc1	BMSC ST2	Promote adipogenic differentiation	2018
Leptin	Primary adipocyte	Promote adipocyte differentiation	2018
AdipoQ AS	Primary adipocyte	Inhibit adipogenesis	2018
OAD	3T3-L1	Promote adipogenic differentiation	2019
CAAlnc1	C3H10	Inhibit adipogenesis	2019
ORA	3T3-L1	Promote adipogenesis	2019
lincADAL	ADSCs	Promote adipocyte differentiation	2019
PGC1β-OT1	ST2 C3H10	Inhibit adipogenic differentiation	2019

	mouse MSCs		
AC092834.1	ADSCs	Inhibit adipogenic differentiation	2020
PVT1	3T3-L1	Promote adipogenic differentiation	2020
HCG11	hAdMSCs	Inhibit adipogenic differentiation	2020
AD	Preadipocyte in chicken	Inhibit adipogenesis	2020
Adi	ADSCs	Promote adipogenic differentiation	2020
MALAT1	C3H10	Promote adipogenesis	2021
LYPLAL1-AS1	ADSCs	Promote adipogenic differentiation	2021
13728	ADSCs	Promote adipogenic differentiation	2021
FR332443	3T3-L1	Inhibit adipogenic differentiation	2021
SAMM50	Buffalo Adipocytes	Promote adipogenic differentiation	2021
RAP2	Preadipocytes	Promote adipogenesis	2022
LIPE-AS1	OP9	Promote adipogenic	2022

		differentiation	
MIR99AHG	3T3-L1	Promote adipocyte differentiation	2022
NR.015556	3T3-L1 C3H10	Inhibit adipocyte differentiation	2022
NEAT1	BMSC	Promote adipogenic differentiation	2022
ACART	3T3-L1	Promote adipocyte differentiation	2023
PLAAT3	Porcine primary adipocyte	Promote adipocyte differentiation	2023
BIANCR	Intramuscular preadipocytes in cattle	Promote adipogenesis	2023

Supplement Table S2

MiRNAs involved in adipogenesis and development

MiRNA	Cell	Function	Year
miR-220a	Yak adipocytes	Promote adipocyte differentiation	2018
miR-23a/27a/24-2 cluster	Bovine adipocytes	Inhibit adipogenesis	2018
miR-27b	3T3-L1	Promote adipocyte differentiation	2019
miR-425	3T3-L1	Promote adipocyte differentiation	2019
miR-130a	BMSCs	Inhibit adipogenic differentiation	2019
miR-16-5p	3T3-L1	Promote adipocyte differentiation	2019
miR-18b-3p	Chicken adipocytes	Inhibit adipocyte differentiation	2019
miR-204	3T3-L1	Promote adipocyte differentiation	2019
miR-107	SGBS	Inhibit adipogenesis	2019
miR-340-5p	Sheep	Inhibit adipocyte	2020

	adipocytes	differentiation	
miR-196b-5p	C3H10<br>ST2<br>BMSCs	Promote adipogenesis	2020
miR-324-5p	Mouse adipocytes	Promote adipocyte differentiation	2020
miR-451	Porcine primary adipocyte	Inhibit adipogenic differentiation	2020
miR-345-5p	3T3-L1	Inhibit adipocyte differentiation	2020
miR-22	3T3-L1	Promote adipocyte differentiation	2021
miR-148a	BMSCs	Promote adipogenic differentiation	2021
miR-222/221	3T3-L1	Promote adipogenesis	2021
miR-370-3p	3T3-L1	Inhibit adipocyte differentiation	2021
miR-410-3p	White preadipocyte	Inhibit adipogenesis	2021
miR-669a-5p	C3H10	Promote adipocyte	2022

	3T3-L1	differentiation	
miR-23b	Porcine preadipocyte	Promote adipocyte differentiation	2022
miR-140	3T3-L1	Promote adipocyte differentiation	2022
miR-214	Duck adipocyte	Promote adipocyte differentiation	2022
miR-26a-5p	Porcine preadipocyte	Inhibit adipocyte differentiation	2023
miR-33a	Bovine adipocytes	Inhibit adipocyte differentiation	2023

Supplement Table S3

CircRNAs involved in adipogenesis and development

CircRNA	Cell	Function	Year
circArhgap5-2	Primary mouse adipocyte	Promote adipogenesis	2019
circFUT10	Bovine adipocyte	Inhibit adipocyte differentiation	2020
circ PLXNA1	Duck adipocyte	Promote adipocyte differentiation	2020
circFLT1	Bovine adipocyte	Promote adipocyte differentiation	2020
circH19	hADSC	Inhibit adipogenic differentiation	2020
circSAMD4A	preadipocyte	Promote adipocyte differentiation	2020
circCDR1as	BMSC	Promote adipogenic differentiation	2020
circHIPK3	HEPG2	Promote adipose deposition	2020
circPTK2	Mouse white preadipocyte	Inhibit adipogenesis	2021
circATXN2	Rat ADSC	Promote	2021

		adipogenesis	
circPPARγ	Bovine adipocyte	Promote adipocyte differentiation	2022
circBDP1	Bovine adipocyte	Promote adipocyte differentiation	2022
circMARK3	3T3-L1 Buffalo adipocyte	Promote adipocyte differentiation	2022
circ0006511	Goat intramuscular adipocyte	Promote adipocyte differentiation	2022
circRNF111	Bovine adipocyte	Promote adipocyte differentiation	2023
circITGB1	Sheep adipocyte	Inhibit adipocyte differentiation	2023
circADAMTS16	Bovine adipocyte	Inhibit adipocyte differentiation	2023
circRBM23	MSCs	Inhibit adipogenic differentiation	2023

*ADSCs: Adipose-derived stem cells; BMSCs: Bone marrow mesenchymal stem cells; ST2, OP9: Murine bone marrow stromal cells; C3H10: Murine mesenchymal stem cell; MSCs: Mesenchymal stromal cells; hAdMSCs: Human adipose-derived mesenchymal stem cells; SGBS: human preadipocytes; HEPG2: Human hepatocellular carcinoma cells.
